# Multifunctional Hydrogels Based on γ-Polyglutamic Acid/Polyethyleneimine for Hemostasis and Wound Healing

**DOI:** 10.34133/bmr.0063

**Published:** 2024-08-05

**Authors:** Xiuyun Li, Wenli Han, Yilin Zhang, Dongmei Tan, Min Cui, Shige Wang, Wenna Shi

**Affiliations:** ^1^ Shandong Provincial Maternal and Child Health Care Hospital Affiliated to Qingdao University, Jinan 250014, Shandong Province, P. R. China.; ^2^ School of Materials and Chemistry, University of Shanghai for Science and Technology, Shanghai 200093, P. R. China.; ^3^ Shandong Cancer Hospital and Institute, Shandong First Medical University and Shandong Academy of Medical Sciences, Jinan 250117, Shandong Province, P. R. China.

## Abstract

Current hemostatic materials have many shortcomings, such as biotoxicity or poor degradability, and do not effectively promote wound healing after hemostasis. To address these limitations, a hemostasis-promoting wound-healing hydrogel, polyglutamic acid/polyethyleneimine/montmorillonite (PPM), comprising polyglutamic acid, 3,4-dihydroxybenzaldehyde-modified polyethyleneimine, and amino-modified montmorillonite (montmorillonite-NH_2_) was constructed in this study. Due to the excellent water absorption abilities of γ-polyglutamic acid, the PPM and polyglutamic acid/polyethyleneimine hydrogels could rapidly absorb the blood and tissue fluid exuded from the wound to keep the wound clean and accelerate the blood coagulation. The homogeneous distribution of montmorillonite-NH_2_ enhanced not only the mechanical properties of the hydrogel but also its hemostatic properties. In addition, the modification of polyethylenimine with 3,4-dihydroxybenzaldehyde provided anti-inflammatory effects and endorsed the wound healing. Cellular and blood safety experiments demonstrated the biocompatibility of the PPM hydrogel, and animal studies demonstrated that the PPM hydrogel effectively stopped bleeding and promoted wound healing. The concept design of clay-based hydrogel may create diverse opportunities for constructing hemostasis and wound-healing dressings.

## Introduction

Uncontrolled bleeding from injuries caused by war, traffic accidents, and natural disasters kills approximately 2 million people annually [1,2]. The major pathological effects of hemorrhage directly increase the incidence of patient complications that can lead to mortality, such as metabolic and cellular dysfunction, hemorrhagic shock, coagulation, and acidosis [3,4]. Typical hemostatic products, such as hemostatic agents, gauze, and bandages, are inadequate for treating large amounts of bleeding and have poor biocompatibility, increasing the risk of secondary damage to the wound during hemostasis. The development of hemostatic products with excellent hemostatic and wound-healing properties would greatly reduce mortality from uncontrolled bleeding [5,6].

Depending on the source, biomedical polymers can be categorized into natural biomedical polymers and synthetic biomedical polymers. The biocompatibility and biodegradability of biomedical polymers make them widely used in hemostatic products, such as hydrogels, sponges, and microspheres [5,7]. Hydrogels are 3-dimensional porous structures that are highly absorbent. Compared with other polymeric biomaterials, hydrogels more closely resemble natural biological tissues [8,9]. Hydrogels formed by crosslinking γ-polyglutamic acid (γ-PGA), which is biodegradable and non-toxic to humans and the environment, are easy to prepare and have excellent properties [10,11]. Moreover, γ-PGA is compatible with healthy and injured skin and has long-lasting moisturizing effects superior to those of hyaluronic acid (HA) and collagen, making γ-PGA a valuable biomaterial for providing nutritive materials to the skin [12,13]. The high water absorption rate of γ-PGA allows it to absorb tissue exudates and thus increase the concentrations of platelets and coagulation factors in wounds [14]. γ-PGA can be crosslinked with the polyamine polyethyleneimine (PEI) through amide bonding to form biomedical hydrogels. Polyamines such as spermine and spermidine interact with DNA and enhance the expression of vascular endothelial growth factor (VEGF) and other endothelial genes associated with cell growth and angiogenesis [15]. Moreover, the hydrophilicity and strong positive surface charge of PEI improve the adherence and aggregation of glial cells, fibroblasts, and other cells to promote wound healing [16,17]. Montmorillonite has been widely used in studies of hemostasis because its swelling properties and negative charge stimulation (activation of blood coagulation) permit a stable hemostasis, unlike other layered silicates [18–22]. However, free montmorillonite may be cytotoxic or induce thrombosis in vivo [23]. Moreover, how to uniformly disperse montmorillonite in the matrix of hemostatic materials is also a challenge. Dopamine derivatives such as 3,4-dihydroxybenzaldehyde (protocatechuic aldehyde [PCA]) with antioxidant and anti-inflammatory effects have good abilities to promote erythrocyte coagulation and vasoconstriction [24–26].

**Fig. 1.**
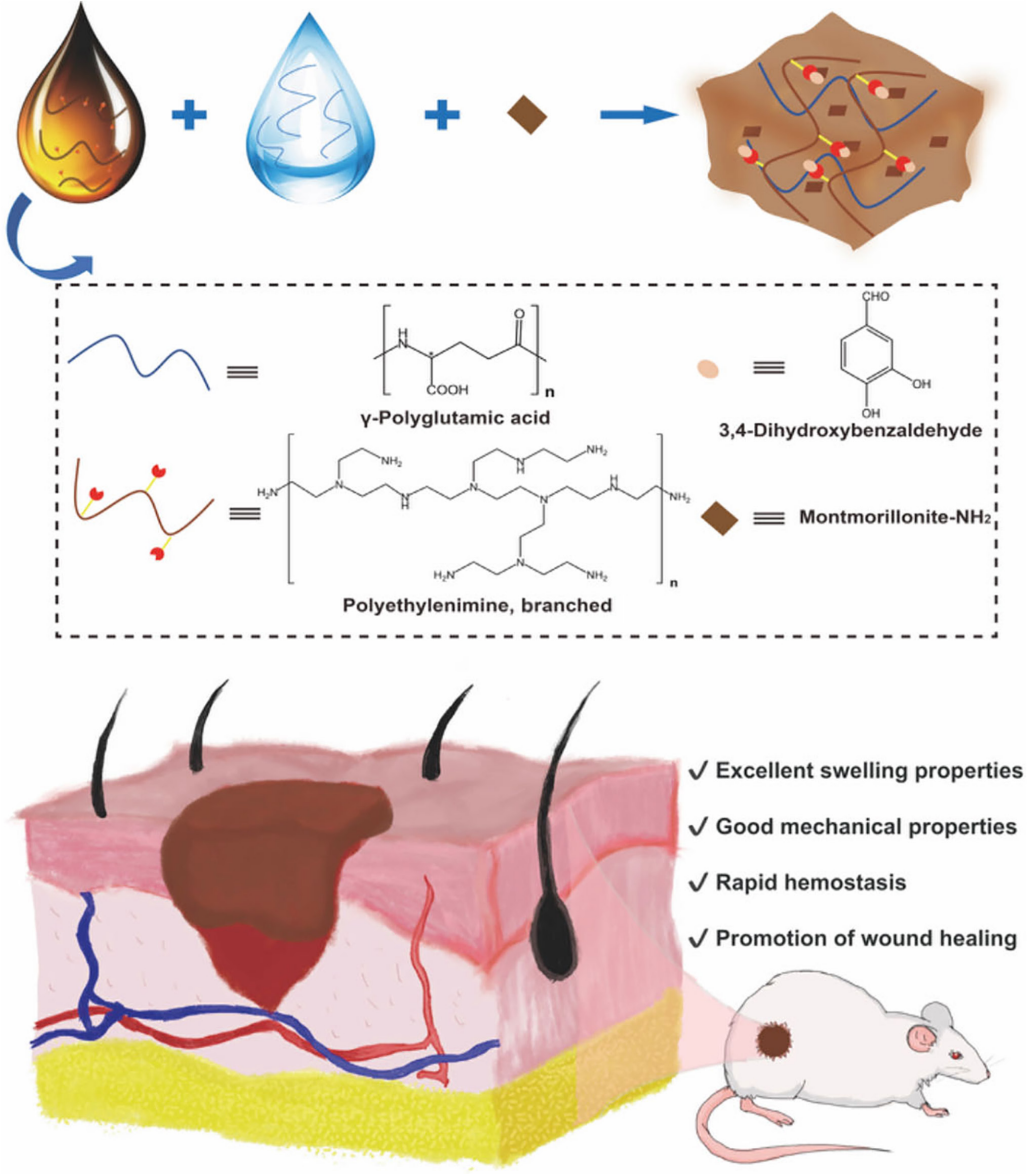
Schematic of the preparation of PPM hydrogel for hemostasis and wound healing.

In this study, montmorillonite-NH_2_ was prepared by modifying montmorillonite with 3-aminopropylethoxysilane (Fig. 1). The resultant montmorillonite-NH_2_ can be conjugated with the carboxyl groups of γ-PGA to improve its dispersion uniformity in the hydrogel system and allow it to be used safely without directly contacting cells. After hemostasis, inflammation at the wound site can inhibit wound healing due to the presence of free radicals. Therefore, we further conferred antioxidant properties on PEI by reacting the aldehyde group of PCA with the amino groups of PEI to synthesize PEI–PCA. Further experiments confirmed the biocompatibility, strong mechanical properties, and antioxidant properties of the polyglutamic acid/polyethyleneimine/montmorillonite (PPM) hydrogel. The hemostasis performance was verified in tail vein bleeding and hepatic injury models. Finally, the hydrogel was applied to promote the healing of whole skin defect wounds in Kunming (KM) mice. This study sheds light on the design of antioxidant and hemostatic hydrogels that are capable of promoting the wound healing.

## Materials and Methods

### Preparation of hydrogels

To prepare PEI–PCA, 5 ml of PEI (Shanghai Myriad, Shanghai, China) was added to 40 ml of deionized water. The solution was stirred well, and 0.375 g of PCA (Shanghai Yuanye Biotechnology Company, Shanghai, China) was added. After stirring at room temperature for 4 h, the solution was dialyzed against deionized water for 5 days and subsequently freeze-dried.

To prepare montmorillonite-NH_2_, 1 g of montmorillonite (Aladdin Reagent Ltd., Shanghai, China) was combined with 5 ml of 3-aminopropylethoxysilane (J&K SCIENTIFIC, Beijing, China) and ground for 15 min in a mortar. The reaction was terminated by the addition of deionized water, and the mixture was washed alternately with ethanol and deionized water and lyophilized to yield the montmorillonite-NH_2_.

To prepare polyglutamic acid/polyethyleneimine/montmorillonite (PPM) hydrogels, 0.09 g of PEI–PCA and 0.45 g of γ-PGA were dissolved in deionized water (3 ml) at room temperature. Next, 0.012 g of montmorillonite-NH_2_ was added with continued stirring. Finally, 0.06 g of N-hydroxysuccinimide (NHS; Aladdin) and 0.06 g of N-(3-dimethylaminopropyl)-N′-ethylcarbodiimide hydrochloride (EDC; Aladdin) were added in deionized water (200 μl) and mixed with the above solution, which was stirred rapidly and subsequently allowed to stand to obtain a hydrogel. Polyglutamic acid/polyethyleneimine (PP) hydrogels were prepared by repeating the above steps without the addition of montmorillonite-NH_2_.

### Characterization of hydrogel components

PEI–PCA and montmorillonite-NH_2_ samples were analyzed by Fourier transform infrared spectroscopy (FTIR) in the range of 4,000 to 400 cm^−1^ on a Nicolet iS20 instrument (Thermo Scientific, USA) to confirm relevant chemical bonding or functional group properties. The mass changes of montmorillonite and montmorillonite-NH_2_ at 30 to 800 °C were observed by differential scanning calorimetry (STA 449 F5, NETZSCH) at a scan rate of 10 °C/min. The microstructures of the PPM and PP hydrogels were compared by scanning electron microscopy (SEM, Zeiss Sigma 300).

### In vitro degradation of hydrogels

The gravimetric method was used to investigate the in vitro degradation properties of the hydrogels. The lyophilized PPM hydrogel was weighed and immersed in 10 ml of deionized water, phosphate buffer solution (PBS), or saline solution at 37 °C in a constant-temperature incubator. Samples were removed at specific time points, washed with deionized water, lyophilized, and weighed. The residual weight ratio was calculated (*W*_0_: the initial hydrogel weight; *W*_t_: the weight of the remaining, Eq. 1):

$$\text{Weight remaining}\;\left(\%\right)=\frac{W_{t}}{W_{0}}\times 100\%$$ (1)

### Antioxidant properties of hydrogels

To study the antioxidant properties of hydrogels, different preparations of hydrogels (15, 25, 50, 100, or 150 mg/ml) were added to a solution of 2,2'-azino-bis(3-ethylbenzothiazoline-6-sulfonic acid) (ABTS), 2-phenyl-4,4,5,5-tetramethylimidazoline-1-oxyl 3-oxide (PTIO), or 1,1-diphenyl-2-picrylhydrazylradical2,2-diphenyl-1-(2,4,6-trinitrophenyl)hydrazyl (DPPH) radicals [27–29] and incubated at 37 °C in an incubator. After removing the hydrogel at the specified time, the absorbance of the supernatant was measured by UV-Vis-NIR spectroscopy (ABTS: 734 nm, PTIO: 557 nm, DPPH: 517 nm) and the supernatant was scanned for wavelength information between 400 nm and 1,000 nm. Meanwhile, a camera was used to record macroscopic images of the color changes of the supernatant during the experiment. The scavenging ratio was calculated according to the following equation (*A*_0_: the initial solution absorbance; *A*_i_: the absorbance measured after incubation, Eq. 2):

$$\text{Scavenging ratio}\;\left(\%\right)=\frac{A_{0}-A_{i}}{A_{0}}\times 100\%$$ (2)

### In vitro hemocompatibility

All animal procedures in this study were approved by the Ethics Committee of the First Affiliated Hospital of Naval Medical University (CHFC(A.E)2023-017). Blood compatibility tests were performed using KM mouse blood provided by Changhai Hospital of Naval Medical University. Erythrocytes were collected by centrifuging the mouse blood at 3,000 rpm for 5 min and washing 3 times with PBS. The erythrocytes were subsequently diluted to 2% in PBS. Next, 2.4 ml of PBS containing 5, 15, 25, 50, or 100 mg of PPM hydrogel was mixed with 600 μl of the 2% erythrocyte suspension. Negative and positive controls were prepared by mixing the 2% erythrocyte suspension with PBS or H_2_O, respectively. The mixtures were incubated at 37 °C in a constant-temperature incubator (2 h). Subsequently, the hydrogel was centrifuged at 3,000 rpm for 5 min. Finally, the absorbance of the supernatant at 541 nm was measured in a UV-Vis-NIR spectrometer (Lambda 25, Perkin Elmer, USA). The hemolytic ratio was calculated based on Eq. 3:

$$\text{Hemolytic ratio}\left(\%\right)=\frac{A_{s}-A_{n}}{A_{p}-A_{n}}\times 100\%$$ (3)

where *A*_s_ is the absorbance of the supernatant from the incubation of erythrocytes with the hydrogel, *A*_n_ is the absorbance of the negative control, and *A*_p_ is the absorbance of the positive control.

### In vivo biosafety assay and degradation

The biosafety of the hydrogels was evaluated by subcutaneously embedding PPM hydrogel blocks (0.2 g, *n* = 3) in male KM mice (6 weeks old, 25–30 g, Shanghai Slaughter Laboratories, Shanghai, China). Each mouse was anesthetized (4% chloral hydrate, intraperitoneal injection) before subcutaneous embedding of the hydrogel. Changes in body weight were determined at specific time points, and the mice were anesthetized and sacrificed at 2, 4, 7, or 14 days after hydrogel embedding. Organs (heart, liver, spleen, lungs, kidneys, and skin) were fixed by paraformaldehyde and stained with hematoxylin and eosin (H&E). Images of the stained organs were acquired by inverted phase-contrast microscopy (Leica DM IL LED, Germany). In addition, blood samples were collected by cardiac puncture to analyze changes in routine blood and blood biochemistry parameters after PPM hydrogel treatment. To investigate the degradation performance of the hydrogel in vivo, the hydrogel blocks embedded under the skin were removed at the time of sacrifice and weighed.

### In vitro whole blood coagulation

Lyophilized hydrogels were preheated at 37 °C for 5 min and subsequently placed in Petri dishes. Sodium citrate was mixed with mouse blood (v:v = 9:1) and dropped on the hydrogel surface (10 μl). Subsequently, 1 μl of 0.2 M CaCl_2_ (prepared from CaCl_2_·2H_2_O, Macklin Biochemicals, Shanghai, China) was added. At 30, 60, 90, 120, or 300 s, deionized (2 ml) water was gently added to suspend the unclotted RBCs. In addition, the coagulation ability of the PPM hydrogel at 300 s was compared with that of the PP hydrogel and cotton gauze. The kinetic curve of whole blood coagulation was plotted for the PPM hydrogel, and the blood clotting index was calculated (*A*_p_: the absorbance of the positive control group [blood treated with deionized water] at 541 nm; *A*_t_: the absorbance of the experimental group at 541 nm, Eq. 4):

$$\text{Blood clotting index}\;\left(\%\right)=\frac{A_{t}}{A_{p}}\times 100\%$$ (4)

Moreover, the above blood samples were tilted at a 30-s interval to observe blood flow to record the clotting time at 37 °C.

### Rat model of hemostatic performance

The hemostatic properties of the PPM hydrogel were evaluated using a severed tail model and a liver volume defect model in male Sprague Dawley (SD) rats (250–300 g, ~10 weeks old, Shanghai Slaughter Laboratories). First, the rats were divided into 3 groups (*n* = 3) randomly and anesthetized by intraperitoneal injection of chloral hydrate (4%). Next, the abdominal cavity was opened with a scalpel blade to expose the liver, and a piece of filter paper was used to collect the blood. After creating a volumetric defect in the liver using the scalpel, the hydrogel (1 g) was quickly placed over the wound. The filter paper was removed for photographs and weighed to calculate blood loss. To establish the SD rat tail-breaking model, the rats were anesthetized and the filter paper was placed under the broken tail. After cutting the rat’s tail into 2 halves with surgical scissors, hydrogel was quickly applied to the severed tail. A piece of filter paper was used to collect the blood. The experimental group was treated with the PPM hydrogel, and the control group was treated with the PP hydrogel. The blank group received no treatment.

### Assessment of wound healing

To investigate the effects of the PPM hydrogel on wound healing, we established 3 groups of whole skin defect model (blank, PP hydrogel, and PPM hydrogel groups, *n* = 6) on the chloral hydrate solution (4%) anesthetized mice. The circular full-thickness open wound with a diameter of 1 cm was made and protected with sterile gauze and bandages after the different treatments. The PP hydrogel (1 g) and PPM hydrogel (1 g) were used to uniformly cover the skin defects in the PP hydrogel group and the PPM hydrogel group, respectively. The wound shrinkage ratio was calculated as follows (*A*_t_: the real-time wound area [*A*_t_ determined using ImageJ]; *A*_0_: the initial wound area, Eq. 5):

$$\text{Wound shrinkage ratio}\;\left(\%\right)=\frac{A_{0}-A_{t}}{A_{0}}\times 100\%$$ (5)

Finally, the wound skin was collected on day 14 to fix in paraformaldehyde (4%). Wound healing was assessed using H&E and Masson staining. The expression of CD31 was evaluated by immunofluorescence staining. To observe lesions, the skin near the wounds was stained with Picrosirius Red. Blood was also collected from the mice, and the serum obtained after centrifugation was used in enzyme-linked immunosorbent assays (ELISAs) to determine the release of epidermal growth factor (EGF), VEGF, and the pro-inflammatory factor tumor necrosis factor (TNF)-α in vivo.

### Statistical analysis

The sample size is 3 (*n* = 3) except as otherwise noted. The statistical results (*P < 0.05, **P < 0.01, ***P < 0.001) of all experiments were assessed by one-way analysis of variance (ANOVA).

## Results

### Preparation and characterization of hydrogels

**Fig. 2.**
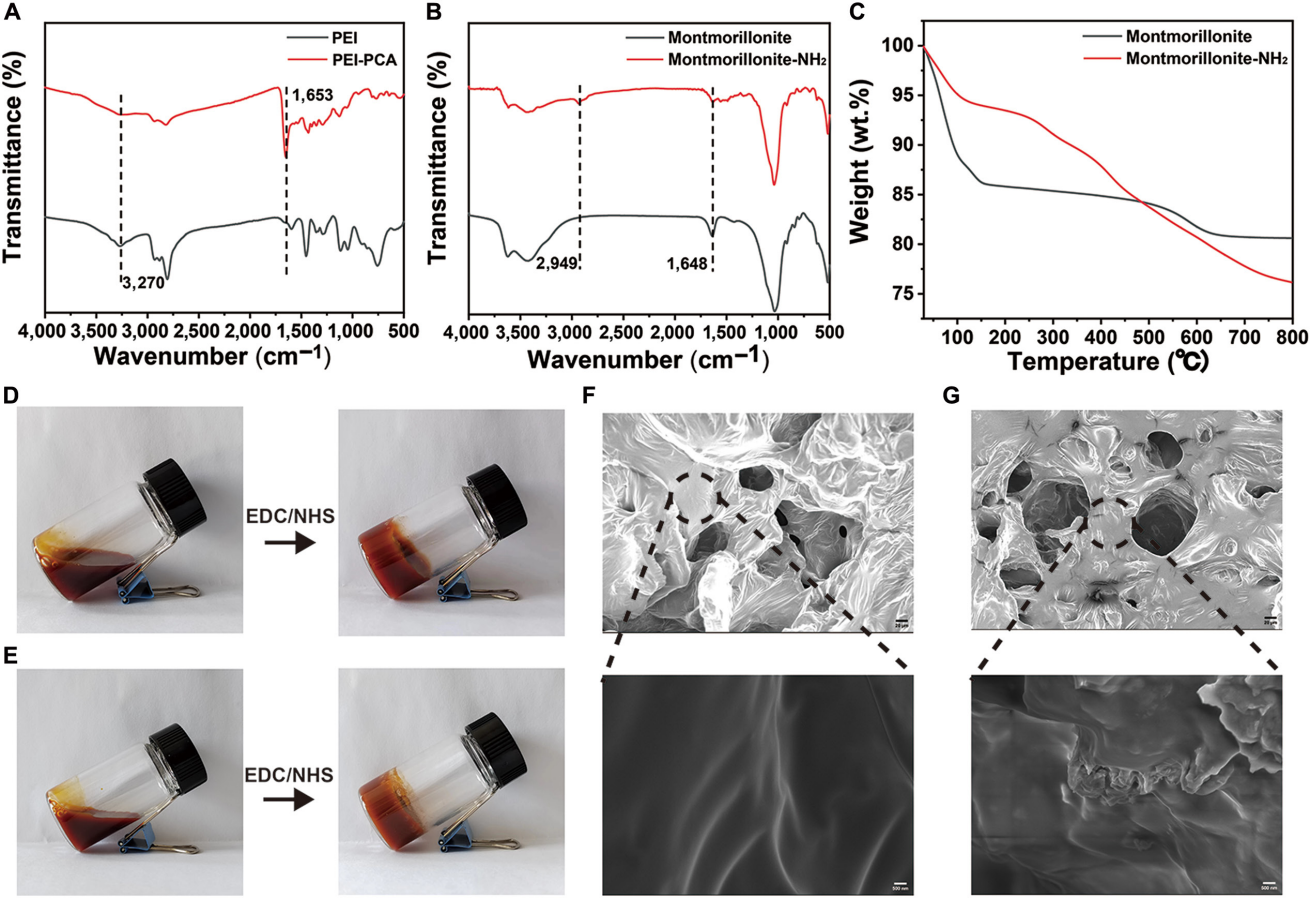
(A) FTIR spectra of PEI and PEI–PCA. (B) FTIR spectra of montmorillonite and montmorillonite-NH_2_. (C) Thermogravimetric analysis of montmorillonite and montmorillonite-NH_2_. (D) Photograph of the solution–hydrogel transition of the PP hydrogel after the addition of the activator EDC/NHS. (E) Photograph of the solution–hydrogel transition of the PPM hydrogel after the addition of the activator EDC/NHS. (F and G) Scanning electron microscopy images of the PP and PPM hydrogels: (F) PP hydrogel, scale bar: 20 μm (top) and 500 nm (bottom); (G) PPM hydrogel, scale bar: 20 μm (top) and 500 nm (bottom).

Before preparing the PPM and PP hydrogels, PEI–PCA was synthesized via a Schiff base reaction between PCA and PEI. FTIR spectroscopy (Fig. 2A) reveals a peak at 1,653 cm^−1^. The FTIR spectroscopy of montmorillonite-NH_2_ (Fig. 2B) exhibits new absorption peaks at 2,870 cm^−1^ and 2,950 cm^−1^ corresponding to the asymmetric and symmetric telescopic vibrational peaks of methylene (-CH_2_-), respectively, and 1,300 to 1,650 cm^−1^ corresponding to C-N absorption. Thermogravimetry further verifies the feasible 3-aminopropyltriethoxysilane grafting of montmorillonite (Fig. 2C). The weight loss of montmorillonite-NH_2_ was 17.5% ± 0.3% over a heating range of 100 to 800 °C, which was attributed to the 3-aminopropyltriethoxysilane decomposition. Finally, the carboxyl group of γ-PGA was activated by EDC/NHS to form an amide bond with the amino group of PEI–PCA, resulting in crosslinking and polymerization to form the PP hydrogel (Fig. 2D). The PPM hydrogel was obtained by doping montmorillonite-NH_2_ into the PP hydrogel (Fig. 2E). The morphologies of the PPM and PP hydrogels after freeze-drying were observed by SEM (Fig. 2F and G). Holes were observed in both hydrogels. The surface of the PP hydrogel was smooth, and after doping, the montmorillonite-NH_2_ was uniformly dispersed on the surface of the PPM hydrogel due to the interaction between the amino group of montmorillonite-NH_2_ and the carboxyl group of γ-PGA.

### Swelling and degradation properties of hydrogels

**Fig. 3.**
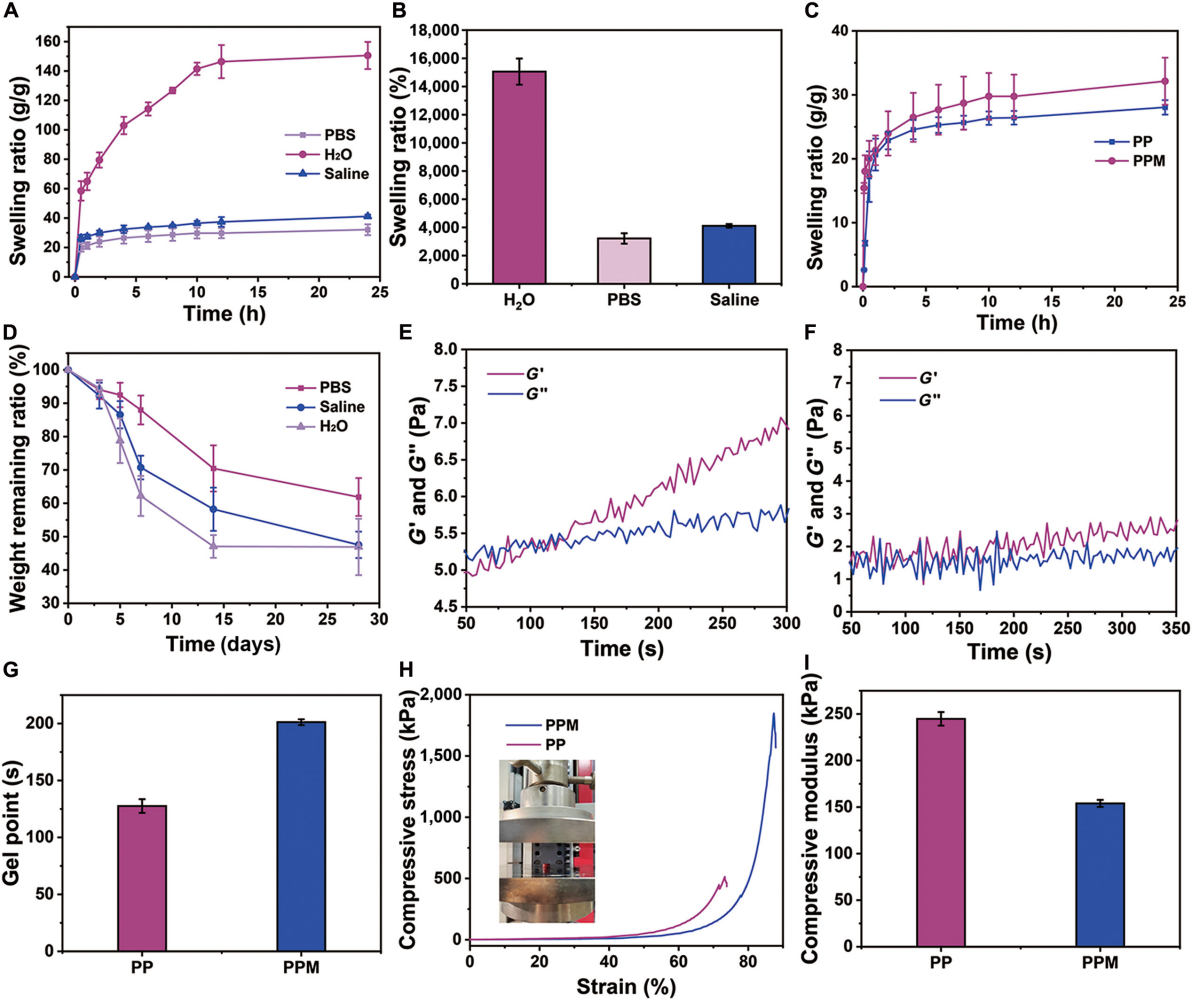
(A) Swelling kinetics curves of PPM hydrogels in H_2_O, PBS, or saline over 24 h. (B) Swelling ratios of PPM hydrogels in H_2_O, PBS, or saline after 24 h. (C) Swelling kinetics curves of PPM and PP hydrogels in PBS over 24 h. (D) Degradation of PPM hydrogels incubated in different solutions for 3, 5, 7, 14, and 28 days. (E and F) Dynamics of the storage modulus (*G*′) and loss modulus (*G*″) after the addition of the activator EDC/NHS to the (E) PP and (F) PPM precursor solutions. (G) Gel points of the precursor solutions of PP and PPM. (H) Compressive stress–strain curves of the PP and PPM hydrogels. (I) Compressive moduli of the PP and PPM hydrogels.

Hydrogels can promote wound healing by absorbing tissue exudates from the wound surface [30–32]. It is important to ensure that a hydrogel does not evoke secondary damage to the wound [33,34]. In particular, the PPM hydrogel reached equilibrium after immersion in deionized water, PBS, or saline for 24 h, with swelling ratios of 15,057.9%, 3,212.9%, and 4,112.7%, respectively (Fig. 3A and B). The swelling ratios of the PP and PPM hydrogels in PBS differed (Fig. 3C). The addition of montmorillonite-NH_2_ to the PP system increased the swelling ratio from 2,931.2% to 3,490.3% (PPM), enhancing the PPM hydrogel’s water absorption capacity. The degradation properties of the PPM hydrogel were assessed after immersion in deionized water, PBS, or saline (Fig. 3D). The PPM hydrogel degraded most quickly in water, consistent with the large swelling ratio. Montmorillonite-NH_2_ decreases the carboxyl group density and weakens intermolecular forces in the hydrogel, thus increasing its susceptibility to degradation. The excellent swelling and degradation properties of the PPM hydrogel support its application in wound hemostasis and healing.

### Rheological and mechanical properties of the hydrogels

To evaluate the rheological properties of the hydrogels, the gelation time and gelation dynamics were evaluated by dynamic time-scanning (Fig. 3E to G). *G*′ of the PP hydrogel exceeded *G*″ at 127 s (Fig. 3E to G), indicating that gelation had occurred. In contrast, the PPM hydrogel reached the gel point at 198 s because the decrease in the density of carboxyl groups prolonged the time required for gelation (Fig. 3F and G). Meanwhile, hydrogel material viscoelasticity was evaluated by phase angle. As shown in Fig. S1, the phase angle of both PPM and PP hydrogels decreases with time, and the state changes from a viscous liquid to an elastomer. However, the trend was more pronounced for PPM, further indicating that doping montmorillonite could enhance the mechanical properties and elasticity of the hydrogel. Compression experiments were performed to assess the mechanical properties of the hydrogels (Fig. 3H). Montmorillonite-NH_2_ increased the compression resistance of the PPM hydrogel (the maximum compressive stress: 1,692.8 ± 141.4 kPa) compared with that of the PP hydrogel. The mechanical duration was also evaluated by determining the compression modulus (Fig. 3I), which provides information on the degree of deformation and elasticity of the material under compressive forces. Water absorption and retention increase as the compression modulus decreases. The compression modulus was 244.8 ± 7.3 kPa for the PP hydrogel and 153.9 ± 3.8 kPa for the PPM hydrogel.

### Antioxidant capacity of the PPM hydrogel

**Fig. 4.**
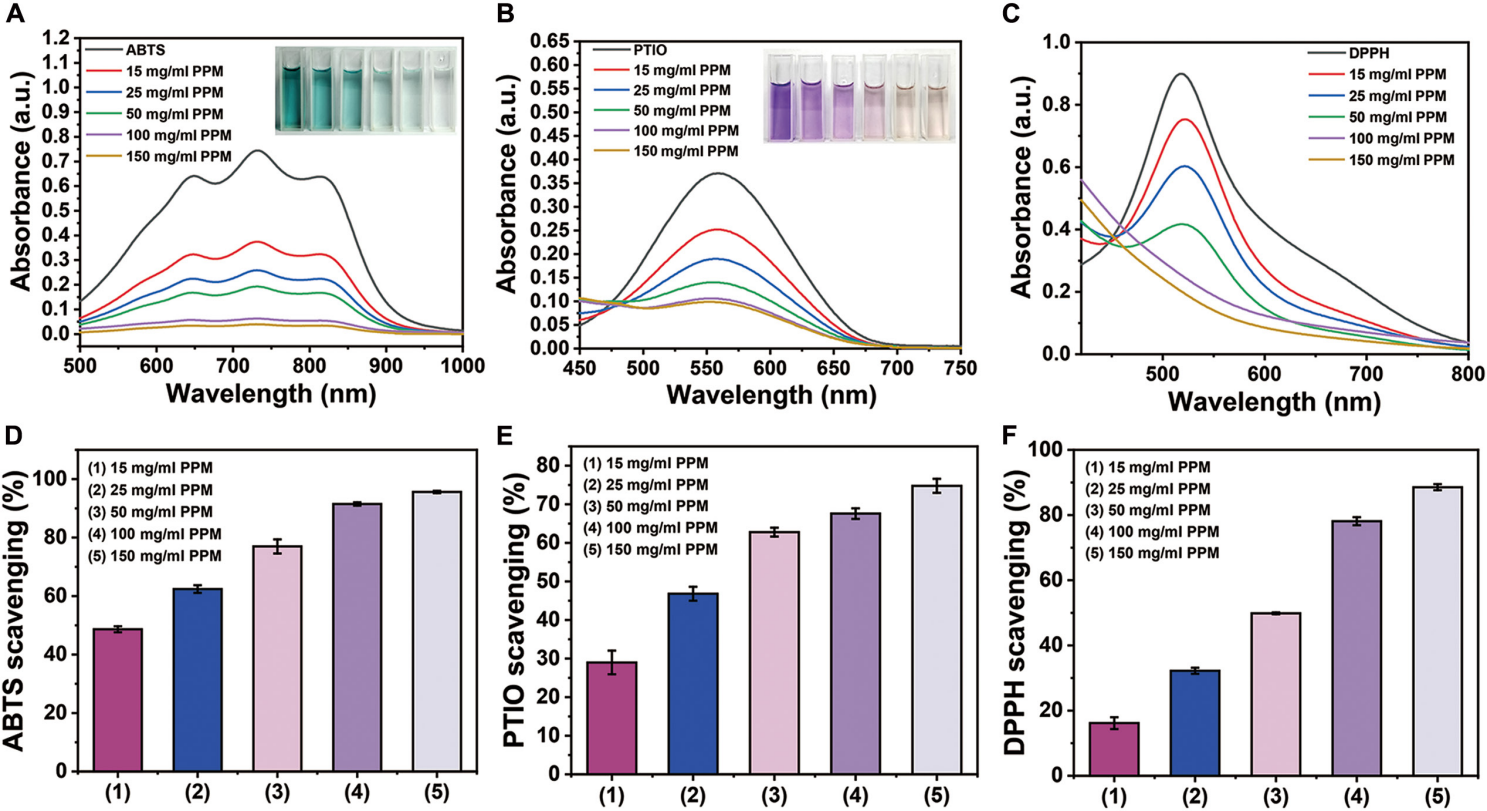
(A) UV–Vis spectra of ABTS radicals after 90 s of removal by different concentrations of the PPM hydrogel. (B) UV–Vis spectra of PTIO radicals after 2 h of removal by different concentrations of the PPM hydrogel. (C) UV–Vis spectra of DPPH radicals after 30 min of removal by different concentrations of the PPM hydrogel. (D) The ABTS radical scavenging ratio at different concentrations of the PPM hydrogel. (E) The PTIO radical scavenging ratio at different concentrations of the PPM hydrogel. (F) The DPPH scavenging ratio at different concentrations of the PPM hydrogel.

A unique characteristic of the PPM hydrogel is that it can scavenge the reactive oxygen species. To validate this point, we evaluated the antioxidant capacity of the PPM hydrogel by assessing its ability to scavenge ABTS, PTIO, and DPPH radicals. The characteristic absorbance of ABTS, PTIO, and DPPH radical solutions decreased and the color faded with the increase of PPM hydrogel solution (from 0 to 150 mg/ml, Fig. 4A to C). To quantificationally examine the antioxidant performance, the ABTS, PTIO, and DPPH radical scavenging ratios were calculated. The results suggested that the ABTS, PTIO, and DPPH radical scavenging ratios were positively correlated with the PPM hydrogel concentration (from 15 to 150 mg/ml), and the maximum ABTS, PTIO, and DPPH radical scavenging ratios were 95.6% ± 0.4% at 90 s, 88.6% ± 0.9% at 30 min, and 74.8% ± 1.8% at 2 h, respectively, when the PPM hydrogel concentration was 150 mg/ml (Fig. 4D to F). After skin injury, excess free radicals often induce severe oxidative stress damage around the wound due to the production of large amounts of reactive oxygen species, impeding wound healing [27].

### In vitro hemocompatibility and cytotoxicity of the PPM hydrogel

**Fig. 5.**
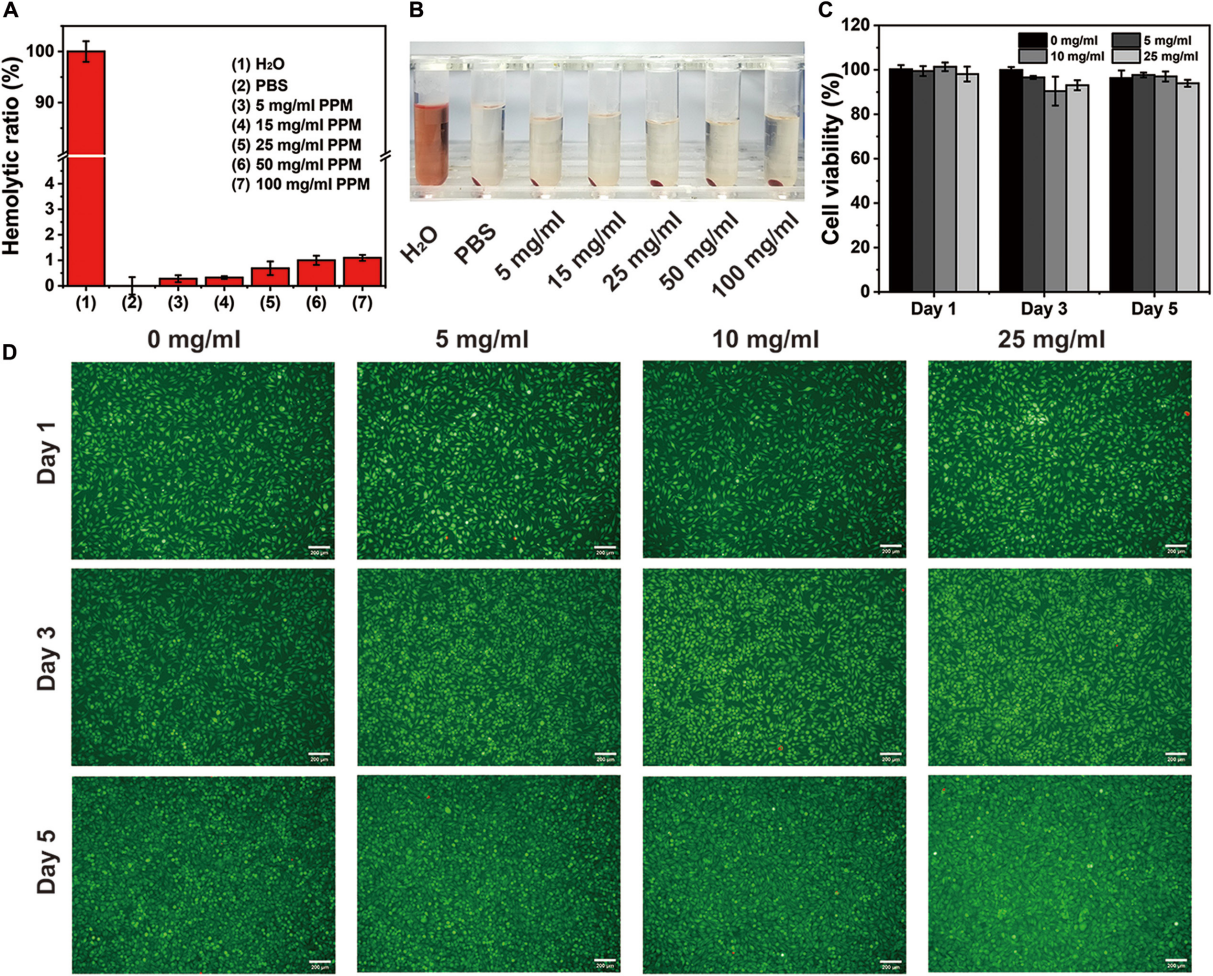
(A) Hemolytic ratios of the PPM hydrogel. (B) Photograph of hemolyzed erythrocytes. (C) Cell viability after 1 to 5 days of treatment with the PPM hydrogel leachate. (D) Live/Dead stained L929 cells treated with the PPM hydrogel leachate (scale bar: 200 μm).

The blood compatibility of the PPM hydrogel was evaluated by assessing the hemolysis of erythrocytes. The hemolytic ratios of the negative and positive controls were 0% and 100%, respectively, and the hemolytic ratios of erythrocytes treated with different concentrations of the PPM hydrogel were 0.28% ± 0.13% (15 mg/ml), 0.3% ± 0.06% (25 mg/ml), 0.7% ± 0.3% (50 mg/ml), and 1.00% ± 0.2% (100 mg/ml), all less than 5% (Fig. 5A and B). To evaluate the cytotoxic effect of PPM hydrogel on cells, we co-cultured L929 cells with different concentrations of PPM hydrogel leachate for 1, 3, and 5 days, and detected the cell viability using the CCK-8 kit [35,36]. The cell survival rate was greater than 95% at all concentrations (Fig. 5C). In addition, the morphology and structure of the L929 cells were qualitatively evaluated by Live/Dead cell staining. Nearly all cells treated with the PPM hydrogel were stained green, with a negligible number of red cells (dead cells, Fig. 5D).

### In vivo biodegradability and safety of the PPM hydrogel

**Fig. 6.**
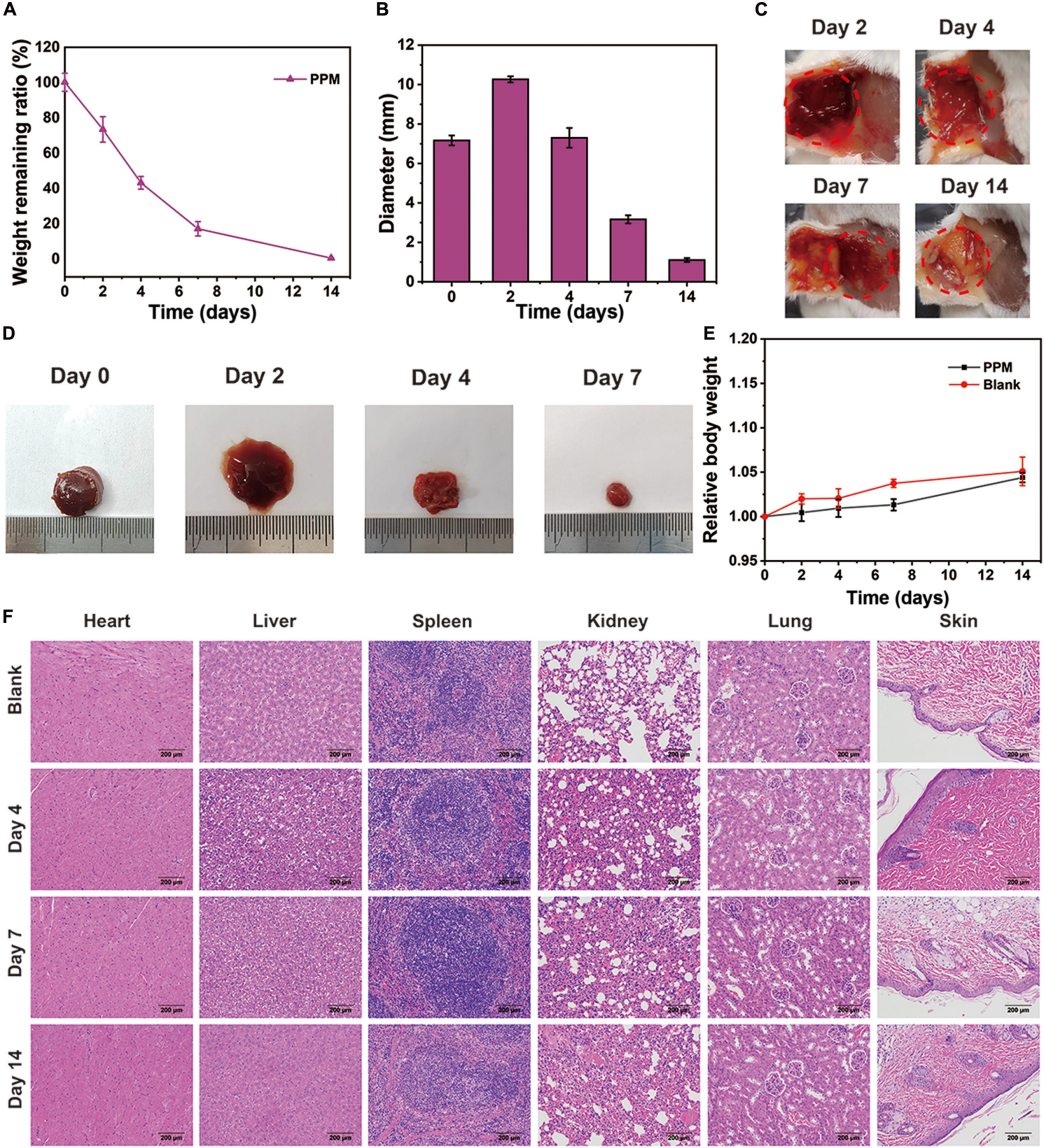
(A) Degradation profile and (B) diameters of PPM hydrogels embedded in mice for 0 to 14 days. (C) Photographs of the appearance of PPM hydrogels embedded in mice for 0 to 14 days. (D) Photographs of changes in the diameter of PPM hydrogels embedded in mice for 0 to 14 days. (E) Changes in the relative body weight of mice treated with PBS (control) or implanted with the PPM hydrogel for 0 to 14 days. (F) H&E staining of vital organs of mice treated with PBS (control) or implanted with the PPM hydrogel for 0 to 14 days (scale bar: 200 μm).

The PPM hydrogel degraded completely within 14 days (Fig. 6A). The diameter of the embedded hydrogel was greatest on day 2, probably due to the absorption of tissue fluid by the hydrogel, which resulted in swelling, softening, changes in shape, and partial degradation (Fig. 6B). After 14 days, almost no residue of the embedded hydrogel remained (Fig. 6C and D). The in vivo safety was assessed by performing in vivo blood safety and histological experiments. Subcutaneous embedding of the hydrogel in mice for 14 days had no important effect on body weight compared with control mice (Fig. 6E). In addition, the blood safety indexes (routine blood parameters and blood biochemistry) were within normal ranges, indicating that the hydrogel had good blood compatibility (Fig. S2). To further verify the safety of the hydrogel, vital organs, including the heart, liver, spleen, lungs, kidneys, and skin section, were removed from mice sacrificed on days 4, 7, and 14 and sectioned for staining (Fig. 6F). No marked differences were observed between mice implanted with the PPM hydrogel and mice in the control group.

### In vitro whole blood coagulation

**Fig. 7.**
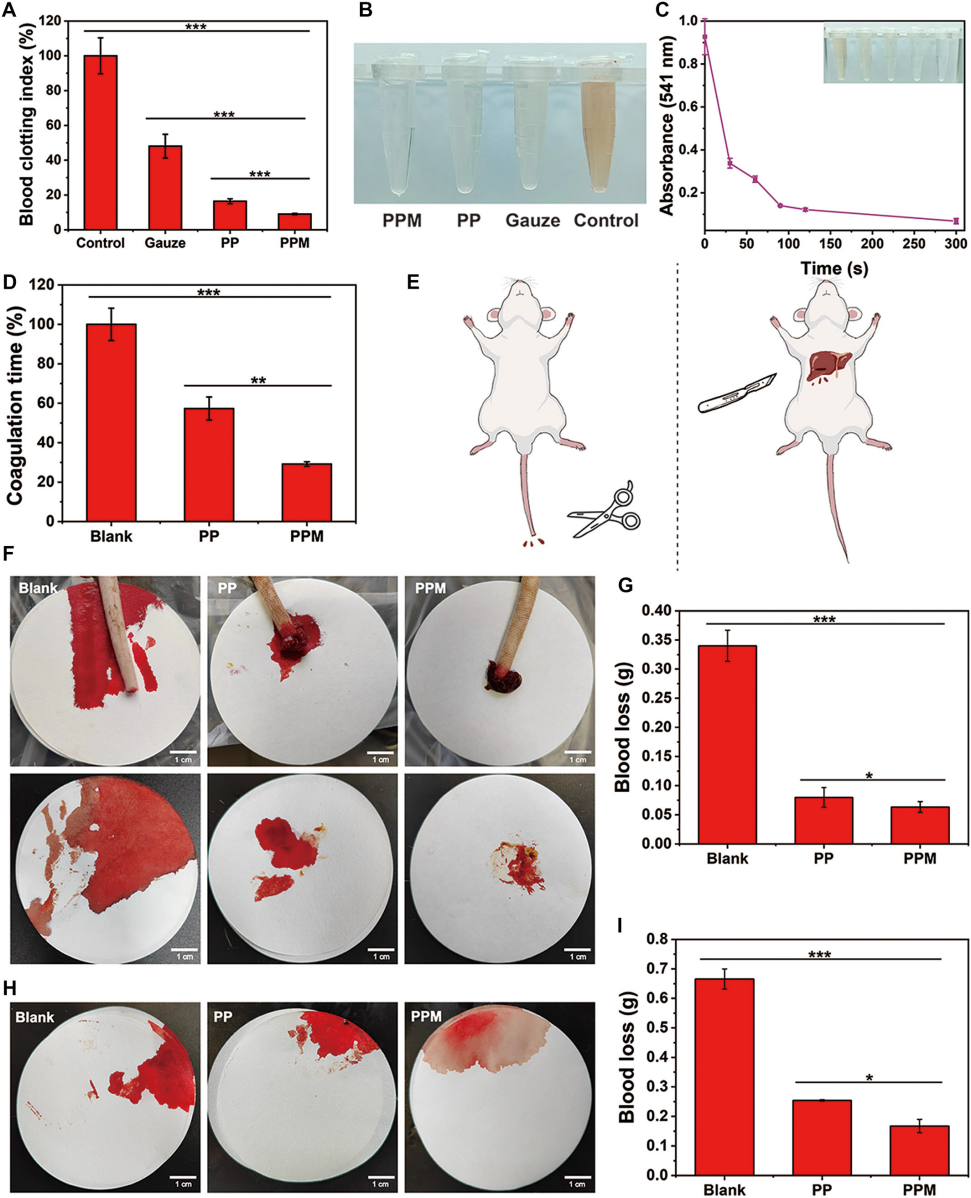
(A) Blood clotting indexes in different treatment groups. (B) Photographs of erythrocyte rupture in whole blood coagulation experiments with the PP hydrogel, PPM hydrogel, and gauze. (C) The kinetic curve of whole blood coagulation by the PPM hydrogel. (D) In vitro clotting times in the different treatment groups. (E) Schematic diagrams of the rat tail vein hemorrhage model and the rat liver injury hemorrhage model. (F) Photographs of blood loss in the rat tail vein hemorrhage model after the different treatments (scale bar: 1 cm). (G) Quantification of blood loss in the rat tail vein hemorrhage model in the different treatment groups. (H) Photographs of blood loss in the rat liver injury hemorrhage model after the different treatments (scale bar: 1 cm). (I) Quantification of blood loss in the rat liver injury hemorrhage model in the different treatment groups.

The in vitro hemostatic and procoagulant properties of the hydrogels were evaluated by measuring the blood clotting index and clotting time. The blood clotting index of the PPM hydrogel was only 9.1% ± 0.4%, significantly lower than those of the PP hydrogel (16.4% ± 1.4%, *P* < 0.001, Fig. 7A and B) and gauze (48.1% ± 6.8%, *P* < 0.001, Fig. 7A and B). Lower blood clotting index values indicate faster coagulation. The absorbance of the PPM hydrogel supernatant at 541 nm was measured at different time points (30 s, 60 s, 90 s, 120 s, and 300 s), and a whole blood coagulation kinetic curve was plotted (Fig. 7C). At 90 s, no distinct red color was observed in the supernatant, indicating that the PPM hydrogel had effectively adhered to the erythrocytes and caused coagulation. The PPM hydrogel coagulated blood in 29.2 ± 1.1 s, whereas the PP hydrogel coagulated blood in 57.3 ± 5.9 s (*P* < 0.01, Fig. 7D).

### Hemostatic properties analysis

A rat tail vein hemorrhage model and a liver injury hemorrhage model were used to examine the hemostatic properties of the PP and PPM hydrogels (Fig. 7E). To construct the rat tail vein hemorrhage model, the tail was cut with surgical scissors to induce bleeding and covered with the hydrogel. After 3 min, the wound was photographed, and blood loss was recorded (Fig. 7F and G). Blood loss was lowest in the group treated with the PPM hydrogel (0.06 ± 0.01 g). Blood loss was 0.08 ± 0.02 g (*P* < 0.05) in the group treated with the PP hydrogel. In both hydrogel-treated groups, blood loss was lower than in the blank group (0.3 ± 0.03 g, *P* < 0.001). In the rat liver injury hemorrhage model, the hydrogel was applied to the defective part of the liver. After stopping the bleeding, the filter paper was removed, photographed, and weighed (Fig. 7H and I). Blood loss was 0.2 ± 0.02 g in the group treated with the PPM hydrogel, 0.25 ± 0.003 g (*P* < 0.05) in the PP hydrogel group, and 0.7 ± 0.03 g (*P* < 0.001) in the blank group (no treatment).

### Wound healing performance evaluation

**Fig. 8.**
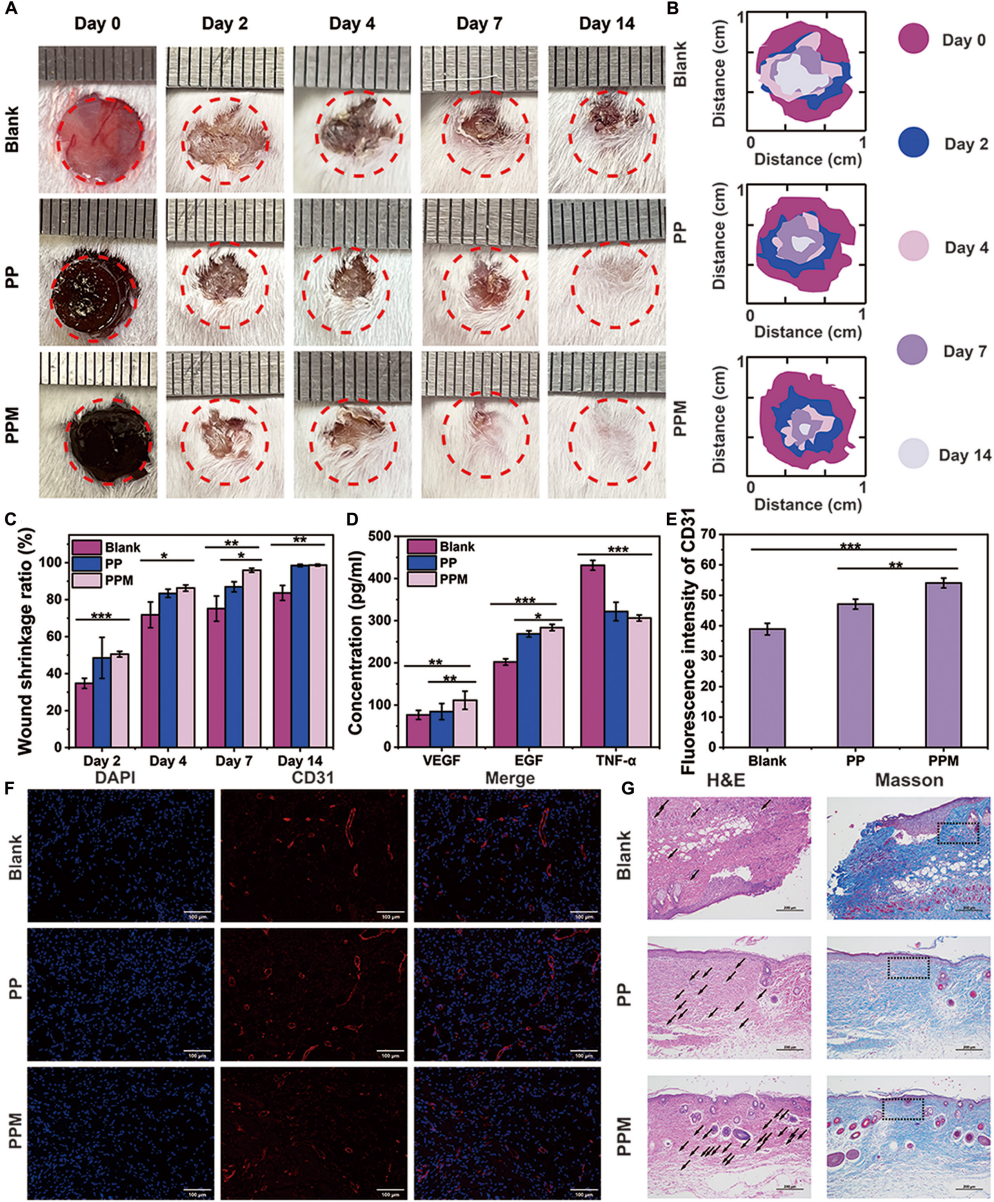
Wound-healing performance in the KM mouse model of total skin defects. (A) Photographs of wounds in different groups after treatments. (B) Simulation of wound healing after different treatments. (C) Wound shrinkage ratio after the different treatments. (D) In vivo concentrations of EGF, VEGF, and TNF-α in the different treatment groups on day 14. (E) Quantitative analysis data of fluorescence staining of CD31 in different treatment groups. (F) Immunofluorescence staining of CD31 in the different treatment groups on day 14 (scale bar: 100 μm). (G) H&E and Masson staining of skin from mice in the different treatment groups on day 14 (scale bar: 200 μm).

Having verified the hemostatic effect of the PPM hydrogel, we subsequently evaluated its therapeutic effect on wounds in a mouse model. All groups exhibited some degree of wound healing after 2, 4, 7, and 14 days (Fig. 8A and B). The wound shrinkage ratios in the PPM and PP groups differed slightly but were greater than that in the blank group on days 2, 4, and 7. On post-treatment days 2, 4, and 7, the wound shrinkage ratio in the PPM hydrogel group was significantly higher than the blank group (day 2: *P* < 0.001; day 4: *P* < 0.05; day 7: *P* < 0.05). On day 7, the wound shrinkage ratio in the PPM hydrogel group was significantly higher than the PP hydrogel group (*P* < 0.05). After 14 days, the wounds healed almost completely in the PPM hydrogel (wound shrinkage ratio: 98.7% ± 0.5%) and PP hydrogel (wound shrinkage ratio: 98.4% ± 0.7%) groups, whereas the wounds in the control group (wound shrinkage ratio: 83.6% ± 4.1%, *P* < 0.01) were still healing and had some scabs (Fig. 8C). To further verify the wound-healing effects of the hydrogels, we quantified the in vivo expression of EGF and VEGF. The up-regulation of EGF expression can activate epithelial cell migration, which facilitates epidermal regeneration. The serum levels of EGF in the PPM and PP hydrogel groups were similar and significantly greater than those in the blank group (Fig. 8D). On day 14, the VEGF concentration in the PPM group was 111.3 ± 21.5 pg/ml, whereas it was much lower in the PP hydrogel group (84.5 ± 19.1 pg/ml) and the blank group (76.5 ± 10.7 pg/ml, *P* < 0.01 versus the PPM group). In particular, the EGF level in the PPM hydrogel group (283.7 ± 7.6 pg/ml) was higher than that in the PP hydrogel group (268.6 ± 7.6 pg/ml, *P* < 0.05) and blank group (202.0 ± 7.5 pg/ml, *P* < 0.001).

Subsequently, we evaluated the effects of the hydrogels on inflammation at the wound site. The PP and PPM hydrogels both reduced the expression of TNF-α due to the presence of PCA (Fig. 8D, *P* < 0.001, versus the blank group). In the blank group, a significant increase in TNF-α expression was observed at 14 days. To further investigate the microscopic effects of the PPM hydrogel, the skin around the wounds was collected for histological analysis. Immunofluorescence staining elucidates that CD31 expression was highest in the PPM hydrogel group and evenly distributed on the skin. For immunocytochemistry, H&E staining verifies that 14 days after treatment, more epithelial membranes, blood vessels, and hair follicles had formed in the PPM group than in the other 2 groups (Fig. 8G, black arrows). In addition, Masson staining of skin revealed the enhanced formation of regular oriented and topologized mature collagen fibers (Fig. 8G, black text boxes) in the PPM hydrogel group. In contrast, the collagen fibers in the control group were loose and parallel to the wound orientation, which might lead to scarring and hyperplasia. Picrosirius Red staining highlights that the yellow or reddish type I fibers in the skin treated with the PPM hydrogel were closely arranged (Fig. S3).

## Discussion

In this research, we constructed a hemostasis-promoting wound-healing hydrogel (PPM) containing γ-PGA, PEI–PCA, and montmorillonite-NH_2_. FTIR confirmed the formation of the Schiff base (C=N) imine bond and the successful synthesis of PEI–PCA. Good water absorption and retention, one of the major desired properties of hydrogel, may make the hydrogel degradation more feasible. Therefore, good swelling and degradation are essential features of hydrogels used as wound dressings. A weight loss comparison between montmorillonite and montmorillonite-NH_2_ evidences that the 3-aminopropyltriethoxysilane accounted for approximately 12.3% of the montmorillonite-NH_2_. Good biodegradability of an implantable material is highly desirable for its potential clinical applications. The PPM hydrogel contained hydrophilic γ-PGA, which has abundant carboxyl groups that form hydrogen bonds with water. As a result, the PPM hydrogel absorbed water quickly and in large quantities. The high osmotic pressures of PBS and saline compared with water reduced absorption by the PPM hydrogel. Rheological and mechanical properties study results outlined that external pressure cannot damage the PPM hydrogel easily, supporting the use of the PPM hydrogel for wound hemostasis. The compression modulus results indicated that the PPM hydrogel had greater water absorption and retention capacity than the PP hydrogel, consistent with the results of the swelling experiments. Good blood compatibility and cell safety will offer great opportunities as an eligible wound hemostatic dressing. The hemolysis study results indicate that the PPM hydrogel does not cause hemolysis or apoptosis of erythrocytes during hemostasis and has excellent blood compatibility. Then, CCK-8 and Live/Dead cell staining results suggested good cytocompatibility of the PPM hydrogel. In vivo degradability and biosafety data highlighted that the PPM hydrogel has the desired degradation characteristics for hemostasis and wound healing, and the PPM hydrogel does not damage organs or cause biotoxicity. Both hydrogels have excellent coagulation properties because PEI effectively adheres to erythrocytes. Moreover, the additional montmorillonite-NH_2_ in the PPM hydrogel adsorbs platelets and endorses thrombus formation for more rapid and effective coagulation.

Wound healing consists of 4 phases: hemostasis, inflammation, proliferation, and remodeling [6]. The PP and PPM hydrogels both contain carboxyl groups that can bind with the blood Fe^3+^ to form a thrombus and accelerate the hemostasis. In addition, γ-PGA has a very high water absorption capacity, endowing the PP and PPM hydrogels with high swelling properties and contributing to effective hemostasis. In the PPM hydrogel, montmorillonite-NH_2_ induces a coagulation cascade in which platelet aggregation promotes blood coagulation, further enhancing the hemostatic effect. Due to the excellent hemostatic properties and water absorption abilities of the PPM and PP hydrogels, blood and tissue fluid exuded from the wound were absorbed rapidly, accelerating coagulation and keeping the wound clean. The excellent water-retention abilities of the hydrogels also helped keep the wound moist and create favorable conditions for wound healing. VEGF is an essential mediator of angiogenesis, another important step in wound healing. Our data suggested that the PPM and PP hydrogels can up-regulate the EGF and VEGF expression, guide cell growth, and accelerate wound healing. When skin is damaged, excess free radicals induce the generation of a large amount of reactive oxygen species, which can damage DNA, protein, and carbohydrates, leading to cellular dysregulation, imbalance between cell growth and apoptosis, and, ultimately, inflammation [37,38]. The results of quantitative analysis of CD31 fluorescence staining were consistent with the fluorescence staining plots, indicating that the PPM hydrogel promoted the angiogenesis. Furthermore, histological results are consistent with the analyses of wound shrinkage and the expression of key proteins, suggesting that the PPM hydrogel promotes wound healing after hemostasis.

In summary, we presented a multifunctional hydrogel containing montmorillonite-NH_2_ to promote hemostasis and wound healing. Montmorillonite-NH_2_ not only enhanced the mechanical resistance of the hydrogel but also facilitated rapid and effective hemostasis. The ligation of PCA to PEI endowed the hydrogel with antioxidant properties that further promoted wound healing. Crosslinking of γ-PGA and PEI–PCA through carboxyl group activation and amide bond formation produced a biocompatible hydrogel with strong water absorption and retention capacity. The PPM hydrogel exhibits good hemostatic properties in the rat tail vein hemorrhage model and the liver defect hemorrhage model. In the mouse whole skin defect model, the PPM hydrogel significantly promoted wound healing, inhibited the secretion of inflammatory factors, increased growth factor expression, and enabled complete healing within 14 days without secondary damage to the wound. What is noteworthy is that this hydrogel has potential for improvement as it currently lacks sufficient weak wet tissue adhesion. This is partly due to the absence of specific functional groups (such as -CHO) that promote tissue adhesion within the hydrogel. Collectively, this research is anticipated to bridge the existing knowledge gap of clay-based hemostatic materials and shed new light on the design of hemostatic and anti-inflammatory multifunctional hydrogels. Furthermore, with the proved antioxidant capacity, the PPM hydrogel can provide a favorable environment for wound healing.

## Data Availability

Data supporting the results of this study can be obtained from the corresponding authors upon request.
