# Supplementary material for: Multifunctional Hydrogels Based on γ-Polyglutamic Acid/Polyethyleneimine for Hemostasis and Wound Healing
Source: Biomater Res. 2024 Aug 5;28:0063. doi: 10.34133/bmr.0063 (PMC11298251; doi:10.34133/bmr.0063)
Supplement: Supplementary 1 — Figs. S1 to S3 [file bmr.0063.f1.docx]

# Supporting information of

# Multifunctional hydrogels based on γ-polyglutamic acid/polyethyleneimine for hemostasis and wound healing

Xiuyun Li,^1,#^ Wenli Han,^2,3#^ Yilin Zhang,^1^ Dongmei Tan,^1^ Min Cui,^1^ Shige Wang,^2,*^ Wenna Shi^3,*^

^1^ Shandong Provincial Maternal and Child Health Care Hospital Affiliated to Qingdao University, No. 9-7 Jiangshuiquan Road, Jinan 250014, Shandong Province, P. R. China.

^2^ School of Materials and Chemistry, University of Shanghai for Science and Technology, No. 516 Jungong Road, Shanghai 200093, P. R. China.

^3^ Shandong Cancer Hospital and Institute, Shandong First Medical University and Shandong Academy of Medical Sciences, No. 440, Jiyan Road, Jinan 250117, Shandong Province, P. R. China.

^#^ These authors contributed equally to this work. * Corresponding email: sgwang@usst.edu.cn (Dr. Wang), and shiwenna7772023@163.com (Dr. Shi)

## *In vitro* swelling studies

The used γ-PGA (≥98%; Mw = 700 kDa) was bought from Shanghai Ekear Biochemical Technology Co. Ltd, Shanghai, China. Two sets of experiments were performed to assess the swelling properties of the hydrogels. First, lyophilized PPM hydrogel samples were weighed and immersed in deionized water, phosphate-buffered saline (PBS), or saline (n = 3). PBS and saline were chosen to simulate the physiological environment. Second, lyophilized PPM and PP hydrogel samples were weighed and soaked in PBS (n = 3) at 37°C in a constant-temperature incubator. Samples were removed from the solution at preset time points and weighed after removing excess liquid on the surface of the hydrogel by absorption by filter paper. The maximum swelling ratio at the swelling equilibrium state was calculated, and the swelling kinetics curve was plotted. The swelling ratio of the hydrogel was calculated by the following equation (W_0_: the initial hydrogel weight; W_t_: the weight of the hydrogel after swelling):

$$Swelling ratio \left( \% \right)=\frac{W_{t}-W_{0}}{W_{0}}\times100\%$$

## Rheological properties of hydrogels

The rheological properties of the prepared hydrogel materials were evaluated using a rotational rheometer. A 200-μL aliquot of PPM or PP hydrogel precursor solution was placed between parallel plates with a diameter of 20 mm and a gap of 1.0 mm. The storage modulus G' and loss modulus G" were analyzed by time-scan tests at 37°C (simulated body temperature) with a constant frequency of 1 Hz and a strain of 1%. Phase angle is the phase difference between stress and strain in an oscillatory deformation and is a measure of the viscoelasticity of a material. If the phase angle is equal to 90^o^, the adhesive is considered to be purely viscous, and conversely, if the phase angle is 0^o^, an ideal elastic solid is indicated. The formula is as follows:

$$Tan(phase angle) =\frac{G"}{G'}$$

## Mechanical properties of hydrogels

The mechanical properties of the hydrogels were evaluated using a universal material testing machine (Zwick Roell Z2.5 TH equipped with a 2.5 kN transducer). A mold was used to prepare cylindrical PP and PPM hydrogels with a diameter of 8 mm and a height of 4 mm. Compressive properties were determined by compressing the hydrogels to 80% of their initial height at a predetermined compressive strain rate of 0.5 mm/min and a strain range of 0%–90%. All tests were performed three times in parallel. The slope of the linear region (40%–50%) of the stress–strain curve was defined as the compressive modulus.

## In vitro cytocompatibility

The *in vitro* cytocompatibility of the PPM hydrogels was evaluated using a mouse fibroblast (L929 cell) model. Mouse fibroblasts (L929) were purchased from the Shanghai Institute of Biochemistry and Cell Biology (Shanghai, China). Sterilized PPM hydrogels were incubated in Dulbecco’s modified Eagle’s medium (DMEM, Corning Ltd, Shanghai, China) at 37 ℃ for 24 h to prepare different concentrations of hydrogel leachate (0, 5, 10, or 25 mg/mL). L929 cells were inoculated into 96-well plates at the same initial density (1×10^4^ cells/well) and cultured for 24 hours. Then, the old medium was replaced with a mixed solution of hydrogel leachate and DMEM and co-cultured with L929 cells for 1, 3 or 5 days. After incubation at constant temperature for 1, 3, or 5 days, survival was assessed using the cell counting kit-8 (CCK-8) assay (Dojindo Laboratories, Japan). In addition, Live/Dead cell staining (Dojindo Laboratories) was performed, and cell morphology was observed by fluorescence microscopy (Leica DM IL, Germany).

## Supplementary figures


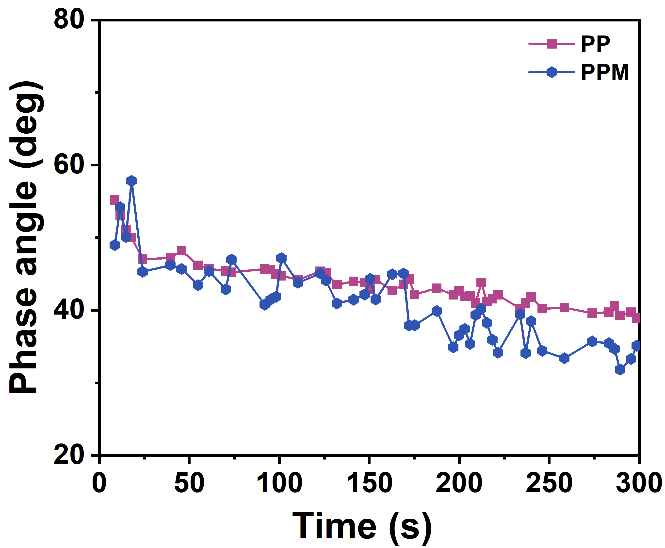


**Fig. S1.** Phase angle curves for PP and PPM hydrogel precursor solutions with activator.


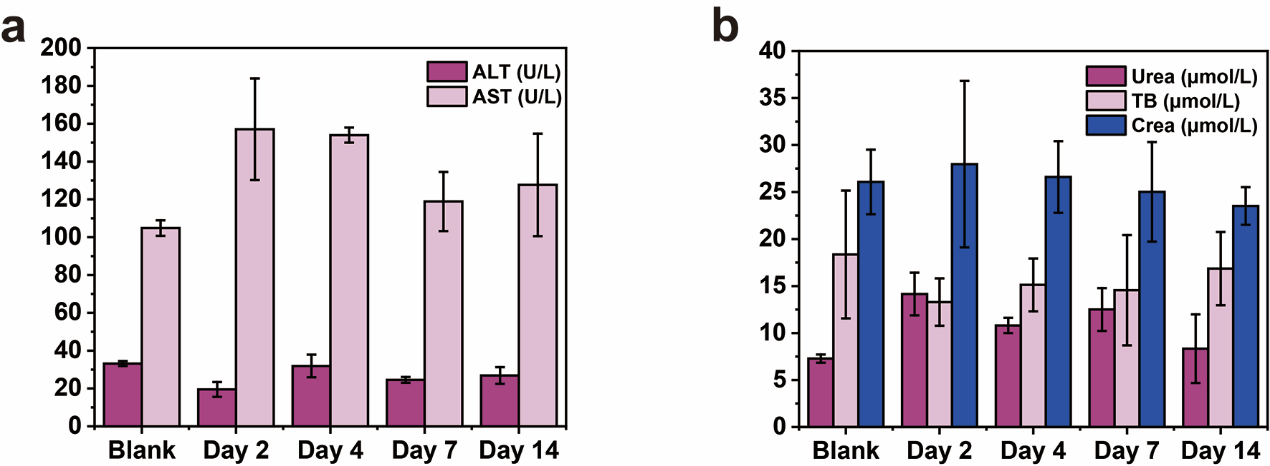


**Fig. S2.** (a-b) Serum biochemical indices in healthy mice (blank group) and mice treated with hydrogel for different days.


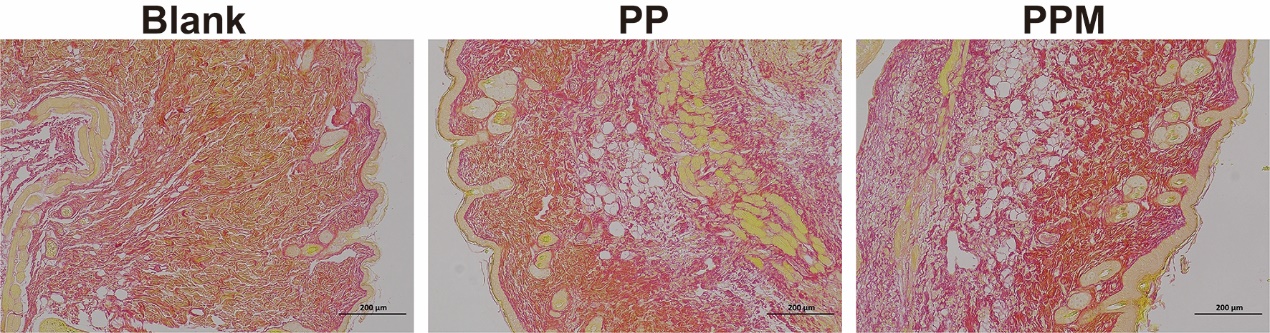


**Fig. S3.** Picrosirius Red staining of skin from mice in the different treatment groups on day 14 (scale bar: 200 μm).
